# Supplementary material for: Transparent soil microcosms for live-cell imaging and non-destructive stable isotope probing of soil microorganisms
Source: eLife. 2020 Nov 3;9:e56275. doi: 10.7554/eLife.56275 (PMC7609051; doi:10.7554/eLife.56275)
Supplement: Transparent reporting form [file elife-56275-transrepform.docx]

***eLife’s* transparent reporting form**

We encourage authors to provide detailed information *within their submission* to facilitate the interpretation and replication of experiments. Authors can upload supporting documentation to indicate the use of appropriate reporting guidelines for health-related research (see [EQUATOR Network](http://www.equator-network.org/%20)), life science research (see the [BioSharing Information Resource](https://biosharing.org/)), or the [ARRIVE guidelines](http://www.plosbiology.org/article/info:doi/10.1371/journal.pbio.1000412) for reporting work involving animal research. Where applicable, authors should refer to any relevant reporting standards documents in this form.

If you have any questions, please consult our Journal Policies and/or contact us: [editorial@elifesciences.org](mailto:editorial@elifesciences.org).

**Sample-size estimation**

- You should state whether an appropriate sample size was computed when the study was being designed
- You should state the statistical method of sample size computation and any required assumptions
- If no explicit power analysis was used, you should describe how you decided what sample (replicate) size (number) to use

Please outline where this information can be found within the submission (e.g., sections or figure legends), or explain why this information doesn’t apply to your submission:

**Fig 3:** For bead analyses, sample size was determined by the number of beads in focus per depth (focal plane), as reported in figure legend.

**Figs 6 & 7:** For single-cell Raman spectra data comparisons, 20 to 30 Raman spectra were collected and analyzed per treatment, in keeping with comparable previous studies comparing Raman spectra of single cells (Berry et al, *PNAS*, 2015).

**Fig 8b:** 30+ Raman spectra were collected and analyzed per distance category, and sample size per subset (CD>0.5 or CD<0.5) was determined with whether CD value of each cell was above or below the threshold.

**Replicates**

- You should report how often each experiment was performed
- You should include a definition of biological versus technical replication
- The data obtained should be provided and sufficient information should be provided to indicate the number of independent biological and/or technical replicates
- If you encountered any outliers, you should describe how these were handled
- Criteria for exclusion/inclusion of data should be clearly stated
- High-throughput sequence data should be uploaded before submission, with a private link for reviewers provided (these are available from both GEO and ArrayExpress)

Please outline where this information can be found within the submission (e.g., sections or figure legends), or explain why this information doesn’t apply to your submission:

Replicate information and outlier treatment information is found in the Methods section for each experiment performed.

**Statistical reporting**

- Statistical analysis methods should be described and justified
- Raw data should be presented in figures whenever informative to do so (typically when N per group is less than 10)
- For each experiment, you should identify the statistical tests used, exact values of N, definitions of center, methods of multiple test correction, and dispersion and precision measures (e.g., mean, median, SD, SEM, confidence intervals; and, for the major substantive results, a measure of effect size (e.g., Pearson's r, Cohen's d)
- Report exact p-values wherever possible alongside the summary statistics and 95% confidence intervals. These should be reported for all key questions and not only when the p-value is less than 0.05.

Please outline where this information can be found within the submission (e.g., sections or figure legends), or explain why this information doesn’t apply to your submission:

-Statistical information (sample size, type of test, p-value) is found in figure legends, or in text within Results section, or both.

-Welch’s t-test was used for all pair-wise comparisons, instead of Student’s t-test which is less robust than Welch’s in cases of unequal variances and non-normal distributions (Delacre, M., et al, *Int’l Rev Soc Psych* 30(1), 2017).

-For multiple comparisons, ANOVAs were performed, followed by Tukey-Kramer HSD (Honest Significant Differences) to correct for multiple testing

-bootstrap confidence intervals were reported in addition to ANOVA and Tukey-Kramer HSD tests; these are particularly informative in cases of highly unequal variances. HSD tests and bootstrap tests were in agreement with each other (i.e. either both falsified or both failed to falsify the hypothesis) in all cases.

-p-values (Welch’s, HSD, or bootstrap) are reported for each test performed.

-95% confidence intervals (95% CI) are presented in figures throughout as measures of variance.

-individual data are presented on top of all boxplots (Fig. 3f, 6abc, 7abc, 8b)

(For large datasets, or papers with a very large number of statistical tests, you may upload a single table file with tests, Ns, etc., with reference to sections in the manuscript.)

**Group allocation**

- Indicate how samples were allocated into experimental groups (in the case of clinical studies, please specify allocation to treatment method); if randomization was used, please also state if restricted randomization was applied
- Indicate if masking was used during group allocation, data collection and/or data analysis

Please outline where this information can be found within the submission (e.g., sections or figure legends), or explain why this information doesn’t apply to your submission:

**Fig 3c:** Beads are embedded in either Nafion or cryolite, and maximum intensities compared (2 groups).

**Fig 3f:** Beads are dispersed in either Nafion or cryolite, and FWHM from coverslip, Nafion at 100 um, and cryolite at 100 um, compared (3 groups).

**Figs 6abc:** Bacteria were treated with either H_2_O or D_2_O, spotted on aluminum slides or dispersed in Nafion or cryolite, and CD values compared (2 groups per substrate).

**Figs 7abc:** Bacteria were treated with either ^12^C- or ^13^C-glucose, spotted on aluminum slides or dispersed in Nafion or cryolite, and Percent ^13^C values compared (2 groups per substrate).

**Fig 8b:** Bacteria from three different microcosms (different biological replicates from different days) were classed into distance categories (“near”, “far”, or “on” hyphae); CD values of different distance categories were compared (3 groups, though ANOVA was also performed on distance AND replicate as variables, see Results).

**Additional data files (“source data”)**

- We encourage you to upload relevant additional data files, such as numerical data that are represented as a graph in a figure, or as a summary table
- Where provided, these should be in the most useful format, and they can be uploaded as “Source data” files linked to a main figure or table
- Include model definition files including the full list of parameters used
- Include code used for data analysis (e.g., R, MatLab)
- Avoid stating that data files are “available upon request”

Please indicate the figures or tables for which source data files have been provided:

Source data provided for all graphs: **Figs 3abc, 3f, 6abcdef, 7abcdef, 8b.** Source microscopy images are also supplied for **Figs 2, 3, 4, 5.**
